# Investigating endophytic fungi of *Calotropis procera* for novel bioactive compounds: molecular docking and bioactivity insights

**DOI:** 10.1186/s12934-025-02710-3

**Published:** 2025-05-08

**Authors:** Sayed M. S. Abo El-Souad, Mohamed A. Dawoud, Mahmoud Ibrahim, Mona M. Soliman

**Affiliations:** Department of Botany and Microbiology, Faculty of Science, Cairo University, Giza, 12613 Egypt

**Keywords:** *Calotropis procera*, Endophytic fungi, Antimicrobial activity, Anticancer activity, 2,2,4,4-Tetramethylpentane, Molecular docking

## Abstract

**Background:**

The rising danger of antibiotic resistance and the increasing burden of cancer worldwide have highlighted the necessity for a constant supply of new antimicrobial drugs and anticancer therapies. Endophytic fungi, recognized as a rich supplier of secondary metabolites with novel bioactivities that have shown promising antimicrobial and anticancer potential, were isolated from the medicinal plant *Calotropis procera*. Approximately 70 segments from the leaves and stems of the *C. procera* plant were evaluated for endophytic colonization, resulting in the isolation and identification of five fungal species based on morphological characteristics.

**Results:**

A total of five endophytic fungal species were isolated from *Calotropis procera* and identified, with *Aspergillus versicolor* exhibiting the highest frequency of occurrence (50%). In contrast, the remaining fungal species were found at a frequency of 25% each. The endophytic fungal filtrates were evaluated for antimicrobial efficacy against seven pathogens, demonstrating significant inhibition zones ranging from 7 to 25 mm. Additionally, the anticancer activity was assessed against two cell lines, MCF-7 and HCT-16, with IC_50_ ranging from 7.8 to 50.4 µg/mL. Among the isolates, the filtrate of *Aspergillus niger* (Accession number PQ568010) exhibited the highest antimicrobial and anticancer activities. The crude extract of *A. niger* was developed to identify the chemical constituents by gas chromatography. The most active component in the extract, as analyzed by ^1^H NMR, revealed that 2,2,4,4-tetramethylpentane was the primary compound responsible for these effects, which demonstrated significant inhibitory activity against *Staphylococcus aureus* and *Bacillus cereus*, with inhibition zones of 23 mm and 20 mm, respectively. Molecular docking studies were performed against Phenylalanine-tRNA ligase alpha subunit of *Bacillus cereus* (UniProt ID: Q633N4), GTPase Der of *Escherichia coli* (UniProt ID: P0A6P5), peptidoglycan-*N*-acetylglucosamine deacetylase of *Listeria monocytogenes* (UniProt ID: A0A3Q0NBH7), DNA gyrase subunit B of *Salmonella typhimurium* (UniProt ID: P0A2I3), Zinc metalloproteinase aureolysin of *Staphylococcus aureus* (UniProt ID: P81177), Agglutinin-like protein 2 of *Candida albicans* (UniProt ID: Q9URQ0), serine/threonine-protein kinase of *Saccharomyces cerevisiae* (UniProt ID: P32600).

**Conclusion:**

The study highlights the potential of endophytic fungi *Aspergillus niger* as a promising source of novel antimicrobial and anticancer agents. The identification of 2,2,4,4-tetramethylpentane as the primary bioactive compound, combined with the molecular docking analyses, provides valuable insights into the mechanisms of action and potential therapeutic applications. These findings underscore the importance of exploring endophytic fungi for the development of new drugs to combat antibiotic resistance and cancer.

## Introduction

An increasing variety of bacterial strains are developing resistance to commonly used antibiotics, including the ESKAPE group of pathogens, which comprises six extremely virulent and antibiotic-resistant bacteria: *Acinetobacter baumannii*, *Enterobacter aerogenes*, *Enterococcus faecium*, *Klebsiella pneumoniae*, *Staphylococcus aureus*, and *Pseudomonas aeruginosa*. Similarly, it is imperative to discover natural antimicrobial compounds. There are renewed efforts to investigate natural compounds as sources of innovative medications. Exploring novel habitats to identify microorganisms capable of producing unique bioactive compounds is critical for addressing the rapidly growing challenge of antibiotic resistance [1]. Additionally, Cancer incidence is expected to rise globally, with over 20 million new cases annually by 2025. According to GLOBOCAN, 14.1 million new cases and 8.2 million cancer-related deaths were reported in 2012. The most frequently diagnosed cancers in Europe include breast, colorectal, prostate, and lung cancer [2]. Chemotherapeutic agents have been widely used for cancer treatment over the past few decades; however, many of them are associated with significant cellular toxicity. Due to these concerns, there has been a growing shift toward natural anticancer compounds derived from endophytic fungi [3].

Endophyte microorganisms, which exist in plant tissues as symbionts, may serve as a source of potential antibiotics [4]. Nevertheless, numerous unique and effective antimicrobials remain undiscovered from these endophytes [5]. These microbes are found in many flora, encompassing trees, herbs, and grasses. New insights highlight their potential to produce highly therapeutic compounds, providing a renewable and environmentally friendly alternative to conventional medical agents [6]. Endophytes develop intricate relationships with their host plants, including parasitism, mutualism, and antagonism, which can lead to the production of valuable secondary metabolites [7].

A wide range of endophytic fungi inhabit the internal tissues of plants in a symbiotic relationship and spend their life cycle inside host plants [8]. In the plant Kingdom, approximately one million species of endophytic fungi are present [9]. Many of these endophytes live within plant tissues without causing infection, leaving the tissues functional and unaffected [10]. Endophytic fungi are an important reservoir of secondary metabolites with various biological activities [11]. These metabolites exhibit diverse bioactivities, including antibacterial, anticancer, antidiabetic, antifungal, antiviral, antioxidant, and insecticidal [12]. Endophytic fungi also produce different bioactive compounds, including enzymes, biocatalysts, and biomolecules utilized across many applications such as industry, medicine, and agriculture. On the other hand, these endophytes help enhance the growth of plants by protecting them from pathogens and relieving abiotic stress [8]. The presence of endophytic fungi inside plant tissues makes an effective barrier preventing any attack by pathogens on the plant [13], where endophyte metabolites inhibit pathogen growth [14]. Many endophytes synthesize novel bioactive secondary metabolites utilized in medicine against different diseases [15]. The natural compounds derived from the endophytic flora of fungi are possible solutions to cancer treatment because they are safe for health, cost-effective, biocompatible, and have fewer toxicity issues. Endophytic fungi secrete a wide range of bioactive compounds, including paclitaxel, podophyllotoxin, camptothecin, vinblastine, hypericin, and diosgenin, which have been isolated from fungal species such as *Thielavia subthermophila*, *Seimatoantlerium nepalense*, *Catharanthus roseus*, *Sinopodophyllum hexandrum*, *Dysosma veitchii*, *Rhizopus oryzae*, *Chaetomella raphigera*, *Aspergillus fumigatus*, and *Seimatoantlerium nepalense*. These compounds exhibit significant anticancer and therapeutic properties, making them promising candidates for drug development [16]. More than half of all known endophytic fungi belong to the phylum *Ascomycota*, followed by *Basidiomycota*. Research on the diversity of endophytic fungi among different plant species has found *Aspergillus*, *Fusarium*, *Penicillium*, and *Piriformospora* as the predominant genera [17]. These bioactive compounds, isolated and purified from various fungal species, have demonstrated effectiveness against multiple cancers, including Kaposi’s sarcoma, prostate, lung, ovarian, and breast cancer. Their anticancer activity is primarily attributed to their ability to induce apoptosis and inhibit cancer progression [18].

*Calotropis procera* is a perennial shrub in the Apocynaceae family that grows primarily in dry and semiarid regions. This plant can be used in vast areas, including feed, medicine, fuel, timber, fiber, textile, and paper phytoremediation, pharmacological uses, and nanoparticle synthesis. It is widely utilized in traditional medicine across North Africa and Asia. Furthermore, high medicinal potential and socioeconomic worth have contributed to its pantropical spread. Morphophysiological modification and resilience to withstand diverse abiotic conditions facilitated its spread beyond the regions where it was initially introduced [19]. *C. Procera* is a well-known plant used traditionally to treat Sinus fistulas, skin disease, somatic problems, and diarrhea [20].

Despite the increasing interest in microbial-derived bioactive compounds, limited studies have focused on endophytic fungi as sources of dual-action agents with both antimicrobial and anticancer properties. This study aims to bridge this gap by isolating and characterizing bioactive compounds from endophytic fungi from *C. Procera*, evaluating their antimicrobial efficacy against multidrug-resistant bacteria and cytotoxic potential against cancer cell lines. Additionally, the bioactive compound structure from the most potent fungal isolate was characterized through various chemical analyses, and its activity was assessed using molecular docking analysis.

## Materials and methods

### Collection of plant samples

Stems and leaves of healthy *Calotropis Procera* plants were collected for endophytic fungal isolation from Giza (29°53′30.8″ N 31°04′13.1″ E), and Cairo (30°03′14.6″ N 31°25′59.9″ E) in Egypt. Samples were collected during summer, with temperatures averaging around 37°C ± 2°C. The collected plant samples were placed in a sterile container to be carried to the laboratory and were processed directly within a few hours.

### Isolation of endophytic fungi from *C. Procera*

According to Mohamed [20], endophytic fungal species were isolated from stems and leaves of *C. Procera*. The collected leaves and stems were washed by running tap water. Subsequently, the surface was sterilized by immersion in ethanol (70%) for 1 min and then 4% sodium hypochlorite for 2 min. Finally, samples were washed with sterile distilled water for 1 min. Stems and Leaves samples air-dried on sterile Whatman no. 1 filter paper to remove excess water. Disinfected plant samples were divided into small segments (5 × 5 mm) by sterile forceps and scalpel. Five sterilized segments were placed upon the surface of each petri dish containing dox agar medium mixed with streptomycin (100 mg/L) to prevent the growth of endophytic bacteria. After 7 days at 28°C, the fungal isolates obtained were purified and transferred to dox agar slants and kept at 4°C for additional experiments.

### Identification of endophytic fungi

The identification of isolated fungal endophytes was based on their morphological characters and microscopic features, followed by the key features [21, 22].

### Preparation of endophytic fungal crude extract

Extraction of endophytic fungal secondary metabolite was prepared by inoculating l00 mL of dox broth medium with a 7-day old culture in 250 mL flask of Erlenmeyer and incubated at 28 °C for 14 days on a shaking incubator at 120 rpm. A total of 35 mL of the organic solvent (ethyl acetate or chloroform) was added to 100 mL of the filtrate in each extraction cycle. This process was repeated three times to ensure thorough extraction. The resulting ethyl acetate and chloroform extracts containing secondary metabolites were collected and concentrated in rotary evaporation. The resulting extracts were kept at 4 °C and examined for their antimicrobial and anticancer activities [23].

### Antimicrobial activity of endophytic fungal crude extract

The crude extract of endophytic fungi was tested for their antimicrobial activity against seven pathogenic microorganisms, three Gram-positive bacteria include *Bacillus cereus* (EMCC 1080), *Staphylococcus aureus* (ATCC 25923), and *Listeria monocytogenes* (ATCC 7644), two Gram-negative bacteria include *Escherichia coli* (wild type strain 93111), and *Salmonella typhimurium* (ATCC 14028), and two fungal species include *Saccharomyces cerevisiae* (ATCC 9763) and *Candida albicans* (EMCC 105). According to the Clinical and Laboratory Standards Institute (CLSI), the antimicrobial activity of endophytic fungal crude extracts was evaluated by the disc diffusion method. The pathogenic bacterial strains were sub-cultured on Luria–Bertani agar plates and incubated at 37 °C for 24 h. In comparison, fungal pathogens strains were sub-cultured on dox agar plates and incubated at 28 °C for 48 h. The tested bacterial and fungal strains were prepared as suspensions with a concentration equivalent to 0.5 McFarland Standard [24]. Finally, a sterile swab was immersed in the prepared suspension and inoculated on Mueller Hinton agar and dox agar plates for bacterial and fungal species, respectively. Under sterile Conditions, Sterilized discs (Whatman no. 5 filter paper, 6 mm diameter) were impregnated with 5 µL of endophytic fungal crude extracts (10 mg/mL DMSO) and placed upon the agar surface. Positive controls were chloramphenicol and fluconazole, while negative controls used were DMSO only. Incubation was carried out at 37 °C for 24 h for bacterial growth and at 28 °C for 72 h for fungal growth. The antimicrobial activities were assessed in millimeters (mm), and the experiment was carried out in triplicate [1, 25].

### Anticancer activity of endophytic fungal crude extract

Cytotoxicity was assessed using the Sulforhodamine B (SRB) assay. Cells (3 × 10^3^/well) were seeded in 96-well plates and incubated with varying concentrations (0, 25, 50, 100 µg/mL) of endophytic fungal crude extract for 48 h. Following fixation with 10% trichloroacetic acid at 4°C, cells were stained with 0.4% SRB for 30 min, washed with 1% acetic acid, and air-dried. The stain was solubilized in Tris base (10 M, pH 10.5), and absorbance was measured at 570 nm using an ELISA microplate reader. IC_50_ values for MCF-7 and HCT-16 cancer cell lines were determined using Prism Software, with BHK cells as the standard control [24].

### Molecular identification of the most potent endophytic fungi

The DNA of most active endophytic fungi was extracted for molecular identification using Quick-DNA™ Miniprep Plus Kit. PCR amplification was carried out using 25 µL My Taq Red Mix, 8 µL DNA template, 1 µL of ITS1 primer (5′-TCCGTAGGTGAACCTGCGG-3′), 1 µL of ITS4 primer (5′-TCCTCCGCTTATTGATATGC-3′), and 15 µL Nuclease-free water [26]. PCR-amplified products were evaluated by electrophoresis and observed by UV light [27]. Sequencing was performed on purified PCR products at GATC Company utilizing an ABI 3730 XL DNA sequencer with forward and reverse primers, integrating conventional Sanger technology. The BLAST tool (NCBI) aligned DNA strands for forward and reverse sequencing. The resulting 18S rDNA sequences of the isolates were blasted against the non-redundant NCBI nucleotide sequence database. The phylogenetic analysis of isolate 18S rDNA sequences was performed to determine the evolutionary relationship between the isolates and reference sequence [28].

### Gas chromatography–mass spectrometry (GC/MS) analysis of bioactive compounds from potent endophytic fungal extract

The potent endophytic fungal crude extract was further analyzed using GC/MS with Agilent 7000E Triple Quadrupole GC/MS. A 1 mL sample was injected into an Agilent 7890A GC system coupled with an HP-5 ms column (5%-Phenyl methyl polysiloxane). The carrier gas utilized was helium at a flow rate of 1 mL/min. The column temperature was set to 38°C. Compounds were detected by comparing mass spectra with NIST and WILEY Libraries [29].

### Separation and purification of bioactive compounds from the potent endophytic fungal extract

#### Thin layer chromatography analysis

Thin Layer Chromatography (TLC) was performed to monitor the identity of crude extract and its fractions and the qualitative purity of the isolated compound. It was also developed to refine the solvent system used for column chromatography. The dried ethyl acetate extract of the most potent endophytic fungi was re-dissolved in ethyl acetate at 20 mg/mL. Analytical TLC was carried out using TLC aluminum plates RP-18 F254 (Merck). Standard chromatograms were prepared by applying a small amount of crude extract to a silica gel TLC plate using capillary tubes and developing it with appropriate solvent systems under saturated conditions. The solvent mixture of ethyl acetate and chloroform in the ratio of 1:1 was employed for the preparative TLC of the extract on TLC plates. Different bands were detected under UV light (254 and 365 nm) and sprayed with a coloring agent (*p*-anisaldehyde). After spraying, the TLC plate was heated to 100℃ until visible colors appeared. Retention factor (Rf) values for all the bands were determined. Subsequently, the silica gel, including the bands, was taken out by scraping and soaked in 1 mL ethyl acetate. After incubating the mixture overnight at room temperature, ethyl acetate portions were separated by centrifugation at 6000 rpm for 10 min and evaporated to complete dryness. To identify the active part with antimicrobial potential, the compounds derived from each band were re-dissolved in 10% DMSO at a concentration of 1000 mg/mL, and antimicrobial activities were assessed again by disc diffusion method against both bacterial and fungal species [29].

#### NMR spectroscopy

The NMR was used to verify the structural elucidation of the most active purified compounds. NMR spectra were obtained at 500 MHz (^1^H NMR) using a JEOL ECA500II spectrometer. Chemical shift results were measured in δ ppm relative to a standard tetramethylsilane (TMS) compound.

### Molecular docking

The three-dimensional crystal structure of a Phenylalanine-tRNA ligase alpha subunit of *Bacillus cereus* (UniProt ID: Q633N4), GTPase Der of *Escherichia coli* (UniProt ID: P0A6P5), peptidoglycan-*N*-acetylglucosamine deacetylase of *Listeria monocytogenes* (UniProt ID: A0A3Q0NBH7), DNA gyrase subunit B of *Salmonella typhimurium* (UniProt ID: P0A2I3), zinc metalloproteinase aureolysin of *Staphylococcus aureus* (UniProt ID: P81177), agglutinin-like protein 2 of *Candida albicans* (UniProt ID: Q9URQ0), serine/threonine-protein kinase of *Saccharomyces cerevisiae* (UniProt ID: P32600) structural protein were retrieved from the UniProt database and prepared using ‘prepare protein’ protocol of BIOVIA Discovery Studio Visualizer 2021. Water molecules were removed, hydrogen atoms were incorporated, and Gasteiger charges were defined via AutoDock tools [30]. The CB-DOCK2 model helped to predict and verify the binding sites of these proteins. The active sites identified were used for protein–ligand interaction studies [31]. The grid boxes for docking were oriented on the expected binding sites. The exhaustiveness parameter was configured to 8, and the default scoring function was used for the docking calculations [32]. The docking was performed between the active binding region of the structure of microbial protein and the tested compound using the Auto-Dock Vina 1.5.7 tool to expect the binding modes and affinities of the tested compound with proteins of microbial species [33]. The binding affinities (ΔG values) in kcal/mol and intermolecular interactions, including hydrogen bonds and hydrophobic interactions, were analyzed and reported as a negative ranking [34].

### Statistical analysis

The antimicrobial activity values of the extracted compounds were reported as the mean ± standard deviation from the triplicate sample. The analysis was carried out by using SPSS (version 21), performing one-way ANOVA, followed by the Duncan test at a significant level of *p* < 0.05 [35].

## Results

### Isolation and morphological identification of endophytic fungi

1. Five fungal species (*Aspergillus flavus*, *A. fumigatus*, *A. niger*, *A. versicolor*, and *Rhizopus arrhizus*) accounting for nine colonies were isolated from leaves and stems of *Calotropis procera* from Giza and Cairo in Egypt (Table 1). Among the five different isolated species, four were classified as species of *Aspergillus*, while the fifth was identified as *Rhizopus*. The morphological and microscopic features of the isolates are shown in Table 2.

**Table 1:** Diversity and counts of endophytic fungi isolated from *C. procera* leaves

Fungal species	Sample 1		Sample 2		Total isolates	Relative density %	Frequency of occurrence %
	Leaf	Stem	Leaf	Stem			
*Aspergillus flavus*	–	–	1	–	1	11.11	25
*Aspergillus fumigatus*	–	–	3	–	3	33.33	25
*Aspergillus niger*	–	–	–	1	1	11.11	25
*Aspergillus versicolor*	2	–	1	–	3	33.33	50
*Rhizopus arrhizus*	1	–	–	–	1	11.11	25
Total count	3	0	4	1	9	100

**Table 2:** The morphological and microscopic features of isolates

	Morphological features		Microscopic features	
*Aspergillus flavus*	Colonies: margin white, yellow green Exudate: lacking Reverse: colorless		Conidiophore: colorless, long, coarsely roughened Conidial heads: radiate Vesicles: globose or flask shaped Conidia: globose to sub-globose, colorless to yellow green	
*A. fumigatus*	Colonies: fast-growing, velutinous, floccose, margin white, greyed-green Reverse: colorless		Conidiophore: short, smooth, gradually enlarging upward, passing imperceptibly into a flask-shaped vesicle, phialides on vesicle directly Conidia: globose to sub-globose, smooth to finely roughened	
*A. niger*	Colonies: black, margin white Exudate: lacking Reverse: colorless to grayed-yellow		Conidiophore: smooth Conidial heads: large, globose to radiate Vesicles: globose or sub-globose Conidia: globose to sub-globose	
*A. versicolor*	Colonies: velutinous, margin white, greenish intermixed with yellowish hyphae Exudate: lacking Reverse: grayed orange		Conidiophore: colorless, smooth Conidial heads: radiate Vesicles: sub-globose to ovate Conidia: globose, echinulate	
*Rhizopus arrhizus*	Colonies: fast-growing, filling the whole plate after 3 days, white at first and becoming greyish		Sporangiophore: smooth-walled, non-septate, unbranched, ends opposite rhizoids Sporangia: white first, then black Sporangiospores: irregular, globose, oval, brownish-black	

### Antimicrobial activity of endophytic fungal crude extract

The tested endophytic fungal crude extract from *C. procera* exhibited antimicrobial potential against seven pathogenic microorganisms, three gram-positive bacteria, including *Bacillus cereus* (EMCC 1080), *Staphylococcus aureus* (ATCC 25923), and *Listeria monocytogenes* (ATCC 7644), two gram-negative bacteria include *Escherichia coli* (wild type strain 93111), and *Salmonella typhimurium* (ATCC 14028), and two fungal species include *Saccharomyces cerevisiae* (ATCC 9763) and *Candida albicans* (EMCC 105) (Table 3). Ethyl acetate and chloroform extract of isolated endophytic fungi showed prominent antimicrobial potential in the inhibition zone against tested bacterial and fungal species.

**Table 3:** Antimicrobial activity of the endophytic fungal crude extract from *C. procera*

Endophytic fungal extracts	Inhibition zone of bacterial sp.					Inhibition zone of fungal sp.		LSD 5%
	*Bacillus cereus*	*Escherichia coli*	*listeria monocytogenes*	*Salmonella typhimurium*	*Staphylococcus aureus*	*Candida albicans*	*Saccharomyces cerevisiae*	
*Aspergillus flavus* (ethyl acetate)	20^c^ ± 2.0	17.67^b^ ± 1.15	17.67^b^ ± 0.58	31^d^ ± 1.0	16.33^b^ ± 0.58	12.33^a^ ± 0.58	10.33^a^ ± 0.58	2.9
*Aspergillus flavus* (chloroform)	17.33^ cd^ ± 0.58	15^bc^ ± 1.73	15^bc^ ± 2.0	30^e^ ± 0	14.67^b^ ± 0.57	9.67^a^ ± 1.53	19.33^d^ ± 1.53	2.8
*Aspergillus fumigatus* (ethyl acetate)	16.67^c^ ± 0.58	14.67^b^ ± 0.58	20.33^d^ ± 0.58	21^d^ ± 1.0	15^bc^ ± 1.0	10.33^a^ ± 1.53	25.67^e^ ± 1.15	2.2
*Aspergillus fumigatus* (chloroform)	13.33^b^ ± 0.58	15.33^bc^ ± 1.53	25.33^e^ ± 1.53	20^d^ ± 1.0	15^bc^ ± 0	10.33^a^ ± 1.53	16^c^ ± 0	2.2
*Aspergillus niger* (ethyl acetate)	16^c^ ± 1.73	20^d^ ± 1.0	24^e^ ± 1.0	20.33^d^ ± 0.58	19^d^ ± 1.0	10.67^a^ ± 0.58	13^b^ ± 1.0	2
*Aspergillus niger* (chloroform)	17.33^c^ ± 2.51	0^a^ ± 0	16^c^ ± 1.0	10.33^b^ ± 0.58	24.33^e^ ± 0.58	26^e^ ± 1.15	20^d^ ± 1.0	3.9
*Aspergillus versicolor* (ethyl acetate)	20^d^ ± 1.0	15^b^ ± 0.58	20^d^ ± 1.0	18.33^c^ ± 1.53	14.67^b^ ± 0.58	10^a^ ± 0	23^e^ ± 1.0	1.9
*Aspergillus versicolor* (chloroform)	17^c^ ± 1.0	14.33^b^ ± 0.58	18^c^ ± 1.0	20^d^ ± 1.0	17.67^c^ ± 0.58	8.67^a^ ± 0.58	25^e^ ± 1.0	2.2
*Rhizopus arrhizus* (ethyl acetate)	15^d^ ± 1.0	17.67^e^ ± 0.58	23^f^ ± 1.0	0^a^ ± 0	16.67^e^ ± 1.15	11.33^c^ ± 0.58	9.67^b^ ± 0.58	3.2
*Rhizopus arrhizus* (chloroform)	12^c^ ± 3.6	10^bc^ ± 1.0	9.67^bc^ ± 0.58	0^a^ ± 0	16.33^d^ ± 0.58	9^bc^ ± 1.0	7.67^b^ ± 2.08	2.2
Fluconazole	0^a^ ± 0	0^a^ ± 0	0^a^ ± 0	0^a^ ± 0	0^a^ ± 0	13.33^c^ ± 0.58	12.0^b^ ± 1.0	2.7
Chloramphenicol	12.67^bc^ ± 0.58	13.33^ cd^ ± 0.58	14.0^de^ ± 1.0	11.67^b^ ± 0.58	15.0^e^ ± 1.0	0^a^ ± 0	0^a^ ± 0	2.9

Antimicrobial activity is represented by the inhibition zone (mm). Numbers expressed as mean ± standard deviation (*n* = 3) for each sample. Different small letters in the same row show mean values at a significant level (*p* < 0.05)

Ethyl acetate extract of endophytic *Aspergillus flavus* showed the highest clear inhibition zone (31 nm) against *S. typhimurium*, followed by *B. cereus* (20 mm). In comparison, the chloroform extract of *A. flavus* was effective against *S. typhimurium* showing an inhibition zone of 30 mm. Ethyl acetate extract of endophytic *Aspergillus niger* exhibited the highest antibacterial activity against *L. monocytogenes* with an inhibition zone of 24 mm. Furthermore, the chloroform extract of *A. niger* showed the highest anticandidal and antibacterial activity against *C. albicans* (26 mm) and *S. aureus* (24 mm). Chloroform extract of endophytic *Aspergillus fumigatus* showed the highest antibacterial activity against *L. monocytogenes* (25.33 mm). In comparison, the highest inhibition zone (26 mm) of *S. cerevisiae* was exhibited by chloroform extract of endophytic *Aspergillus versicolor*. In the present investigation, *S. typhimurium* was more resistant to ethyl acetate and chloroform extract of *Rhizopus arrhizus*.

### Anticancer activity of endophytic fungal crude extract

**Fig. 1:**
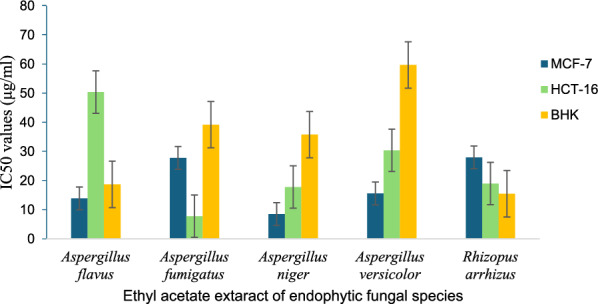
Cytotoxic activity of endophytic fungal extracts derived from *C. procera* against three cell lines: MCF-7 (breast cancer), HCT-16 (colon cancer), and BHK (standard cell). The IC50 values, representing the drug concentrations that inhibited 50% of cell proliferation, were determined for each cell line

The ethyl acetate extracts of the five endophytic fungal species exhibited in vitro cytotoxicity activity against the two cancer cell lines: MCF-7 (Breast cancer) and HCT-16 (Colon Cancer). At the same time, BHK was used as a standard cell line (Fig. 1). The crude extract of the endophytic fungus *Aspergillus niger* exhibited the highest anticancer activity against MCF-7 and HCT-16 with an IC50 value of 8.5 and 17.8 µg/mL, respectively. The ethyl acetate extract of the endophytic *Aspergillus fumigatus* displayed strong cytotoxic activity against HCT-116, exhibiting an IC50 value of 7.8 µg/mL. On the other hand, crude extracts of *Aspergillus flavus* and *Rhizopus arrhizus* exhibited cytotoxic effects against cancer cells, but only at concentrations toxic to normal line cells. The crude extract of the endophytic fungus *Aspergillus versicolor* exhibited anticancer activity against MCF-7 and HCT-16 with an IC50 value of 15.6 and 30.4 µg/mL, respectively. In our study, *Aspergillus niger* was chosen for further investigation due to its superior antimicrobial and anticancer activities.

### Molecular identification of the most potent endophytic fungi

**Fig. 2:**
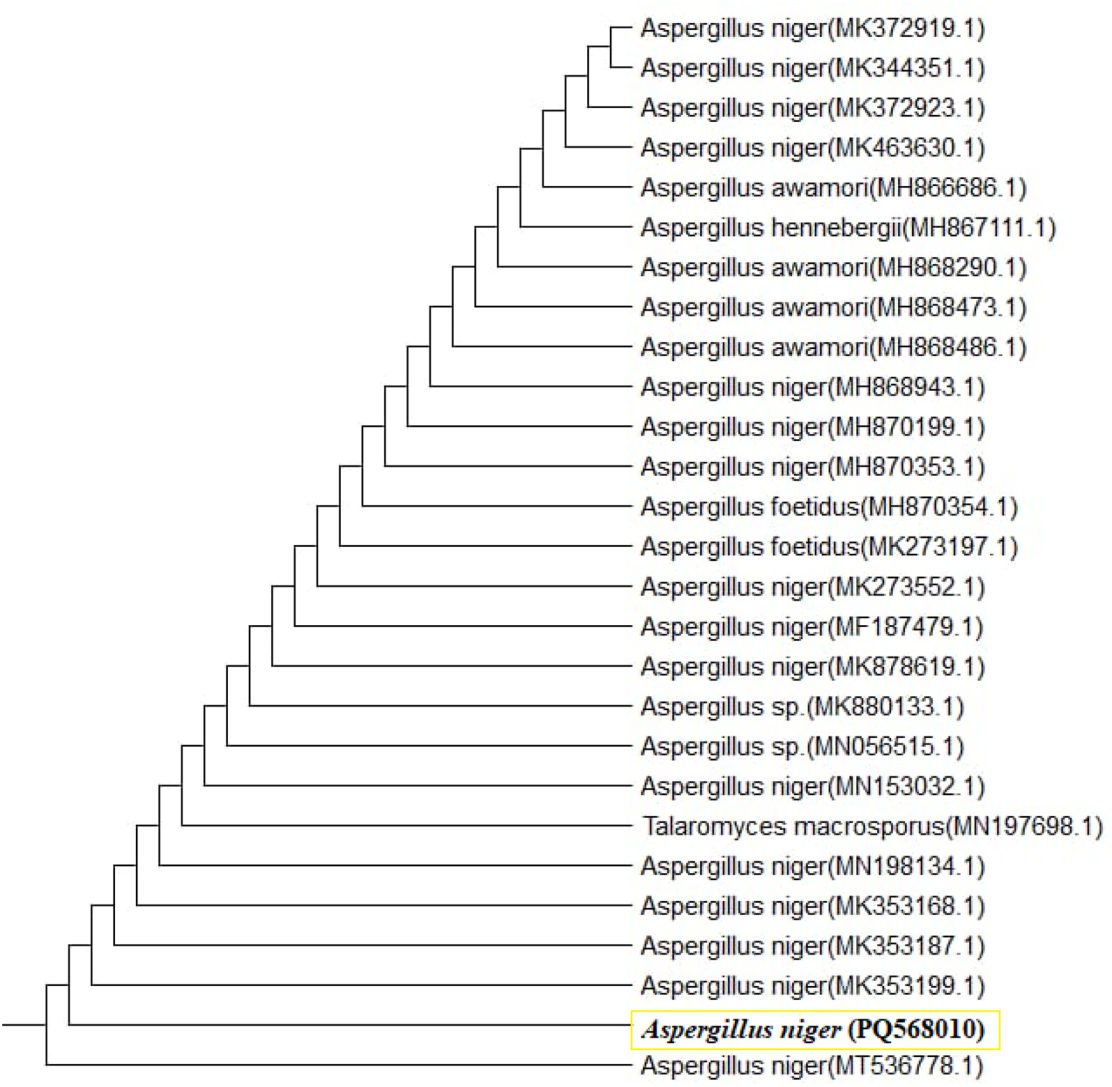
Phylogenetic tree of the most active fungal strain, *Aspergillus niger*, showing its relationship with closely related species. The strain is identified with the accession number PQ568010

Alignment of sequences of the tested isolated *Aspergillus niger* based on their ribosomal internal transcribed spacer (ITS) sequences showed 100% sequence homology with *A. niger* isolate cu-17 (MT536778.1) from the gene bank based on the phylogenetic analysis, and it has accession number PQ568010. Also, the sequence and its evolutionary relationship with the other related fungal species were illustrated by the phylogenetic tree presented in Fig. 2.

### Gas chromatography–mass spectrometry (GC/MS) analysis of bioactive compounds from potent endophytic fungal extract

The ethyl acetate extract of the tested isolated *Aspergillus niger* was characterized and identified by GC/MS analysis. GC/MS analysis revealed that the fungal endophyte *Aspergillus niger* can synthesize a variety of bioactive compounds (Table 4). Various types and numbers of compounds were detected by GC–MS study. *A. niger* was the most effective endophytic fungal species, which synthesizes secondary metabolites, including polyethylene polymer, 2,2,4,4-tetramethylpentane (Nonane), propyl hexadecanoate (propyl palmitate), and stearamide.

**Table 4:** Compounds of crude extract of *A. niger* detected by GC/MS analysis at different retention times

Name of compounds	Molecular formula	Retention time (min)	Area % (cm^2^)
Polyethylene polymer	(C₂H₄)_*n*_	73	30
2,2,4,4-Tetramethyl pentane (nonane)	C_9_H_20_	15	18
Propyl hexadecanoate (propyl palmitate)	C_14_H_28_O_2_	27	15
Stearamide	C_18_H_37_NO	51	12

### Separation and purification of bioactive compounds from the potent endophytic fungal extract

#### Thin layer chromatography analysis

**Fig. 3:**
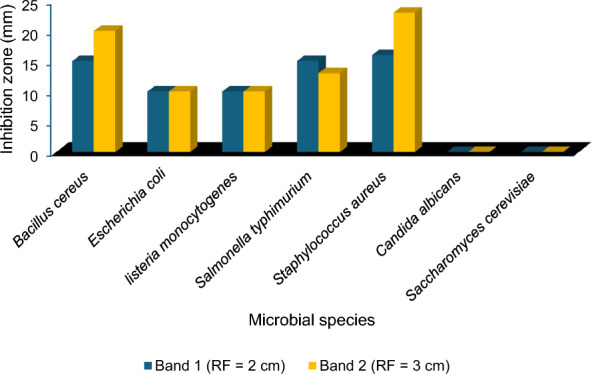
Antimicrobial activity of 2 distinct bands of ethyl acetate extract of *A. niger* identified through thin layer chromatographic (TLC) analysis. Antimicrobial activity is represented by the inhibition zone produced by active fractions against the tested microbial species

The antimicrobial metabolites from the ethyl acetate extract of *A. niger* were separated and partially purified using the TLC method. The ethyl acetate extract of *A. niger* showed two bands with different Rf values, including band 1 (R_f_ = 2 cm) and band 2 (R_f_ = 3 cm) under UV light. Band 2 (R_f_ = 3 cm) showed significant inhibitory activity against *Staphylococcus aureus* and *Bacillus cereus* with inhibition zones of 23 and 20 mm, respectively, while band 1 showed the highest inhibitory efficacy against *Salmonella typhimurium* with an inhibitory zone of 15 mm (Fig. 3).

#### Nuclear magnetic resonance (NMR) analysis

**Fig. 4:**
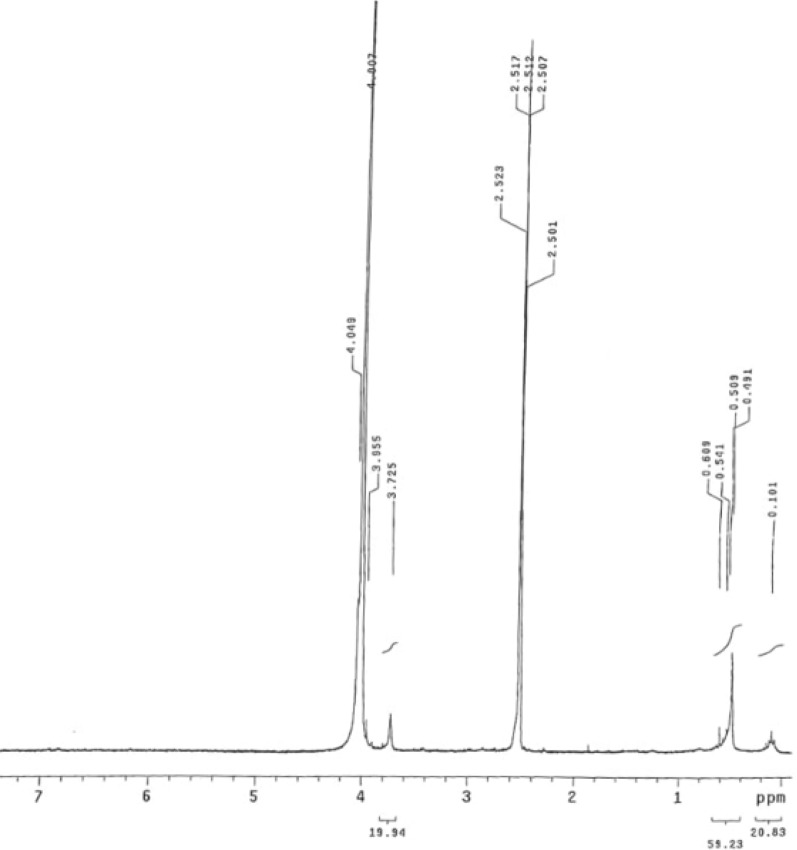
^1^H-NMR of the compound in ethyl acetate extract of *A. niger* from the second band (R_f_ = 3 cm) from TLC

**Fig. 5:**
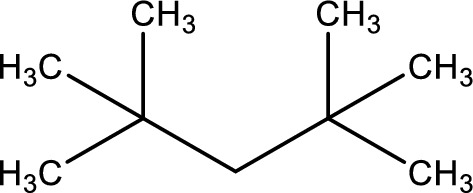
2,2,4,4-Tetramethylpentane

^1^H NMR analysis of the antimicrobial compound isolated from band 2 of TLC indicates a molecular formula of C₉H₂₀, suggesting a highly branched alkane structure (Fig. 4). A single peak at δ 0.1 ppm corresponds to tetramethylsilane (TMS), used as an internal reference peak. The existence of the four peaks at δ 0.491, 0.509, 0.541, and 0.609 ppm is attributed to terminal methyl (–CH₃) groups. In addition, a multiplet at δ 2.501, 2.507, 2.512, 2.517, and 2.523 ppm is assigned to H on carbons near a branching point in the alkane chain. The peaks at δ 3.725, 3.955, 4.007, and 4.049 ppm suggest H on methylene (–CH_2_) groups adjacent to the central carbon atom. Based on the molecular formula and the NMR spectral data, the compound is likely a highly branched alkane, with 2,2,4,4-tetramethylpentane being a potential structural candidate (Fig. 5).

### Molecular docking

Modeling studies were conducted to elucidate the binding model and interaction of the bioactive compounds from the potent endophytic fungal extract (2,2,4,4-tetramethylpentane) within the target protein’s active site. Accordingly, molecular docking studies were carried out against five bacterial proteins and two fungal proteins, including Phenylalanine-tRNA ligase alpha subunit (UniProt ID: Q633N4), GTPase Der (UniProt ID: P0A6P5), peptidoglycan-*N*-acetylglucosamine deacetylase (UniProt ID: A0A3Q0NBH7), DNA gyrase subunit B (UniProt ID: P0A2I3) and Zinc metalloproteinase aureolysin (UniProt ID: P81177), which are expressed for *Bacillus cereus*, *Escherichia coli*, *Listeria monocytogenes*, *Salmonella typhimurium* and *Staphylococcus aureus*, respectively. However, Agglutinin-like protein 2 (UniProt ID: Q9URQ0) and serine/threonine-protein kinase (UniProt ID: P32600) are expressed for *Candida albicans* and *Saccharomyces cerevisiae*, respectively. Proteins were optimized by eliminating water molecules, adding polar hydrogens, and assigning Gasteiger charges. The bond order of the ligand and protein was adjusted. The bioactive compound from the potent endophytic fungal extract was obtained from the PubChem database in SDF format and then subjected to energy minimization by Avogadro software.

**Fig. 6:**
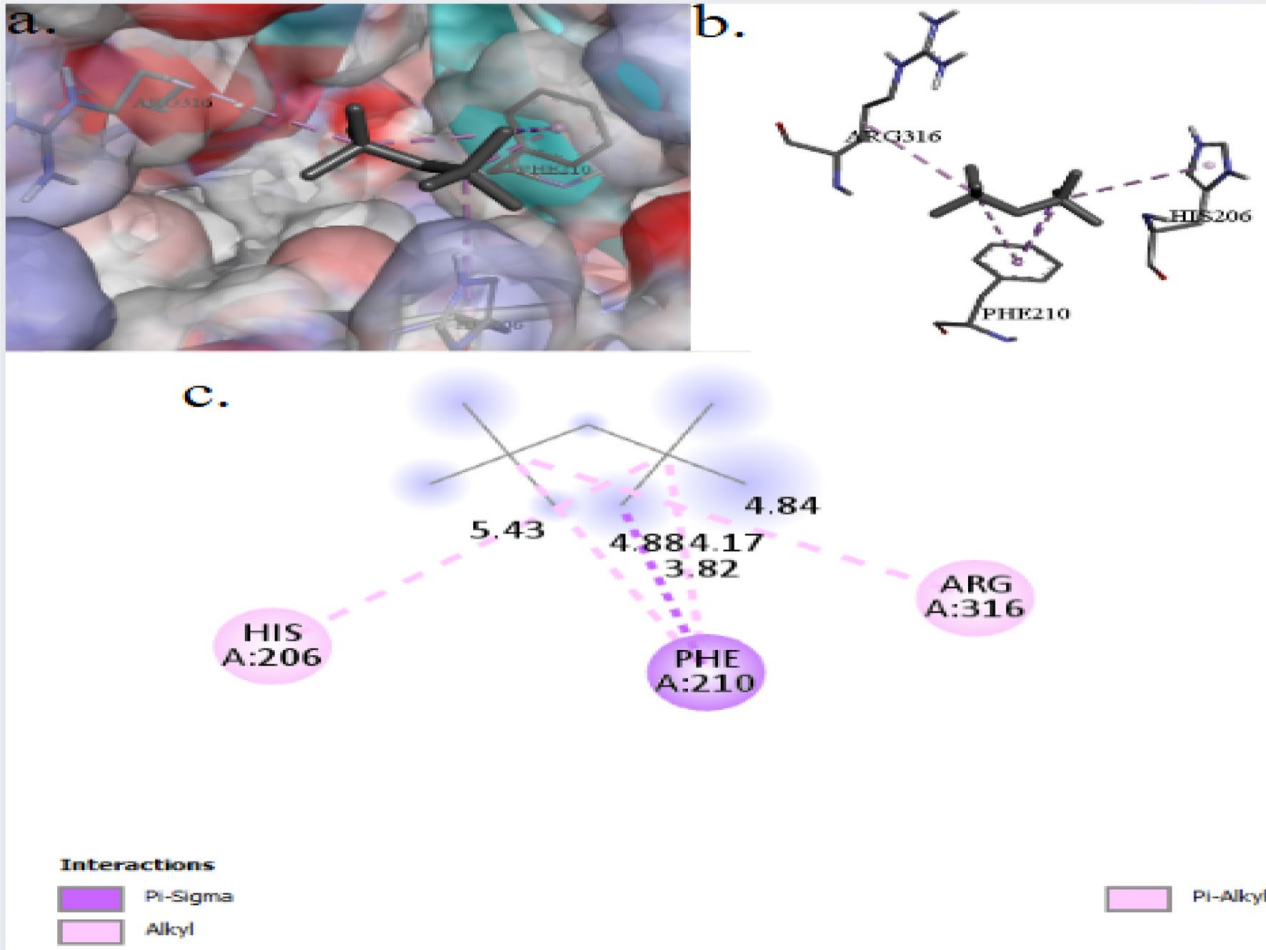
3D (**a**, **b**) and 2D (**c**) molecular docking interaction between the extracted 2,2,4,4-tetramethylpentane and the active site of Phenylalanine-tRNA ligase alpha subunit (UniProt ID: Q633N4) of *Bacillus cereus*

**Fig. 7:**
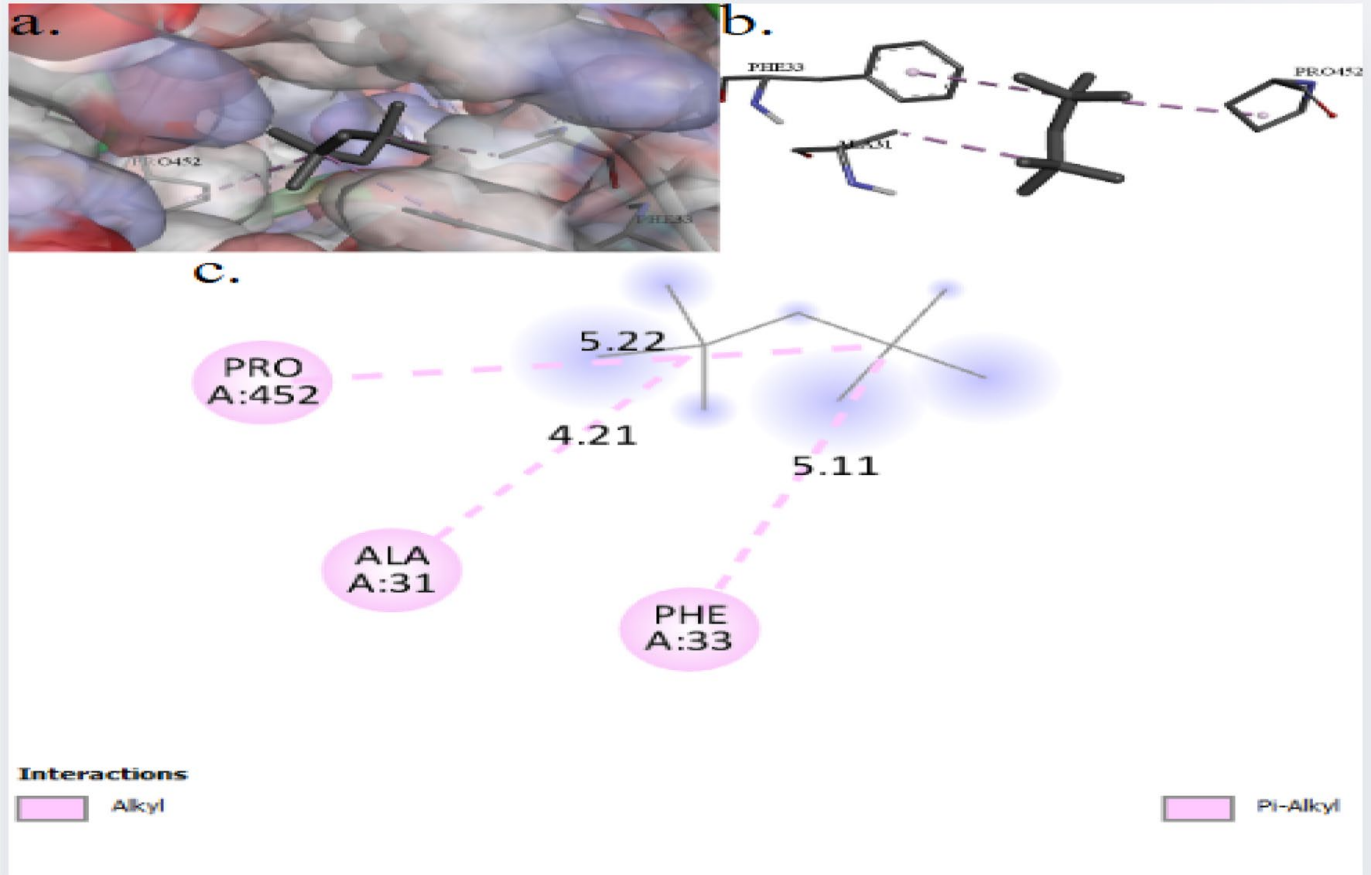
3D (**a**, **b**) and 2D (**c**) molecular docking interaction between the extracted 2,2,4,4-tetramethylpentane and the active site of GTPase Der (UniProt ID: P0A6P5) of *Escherichia coli*

The binding interactions of 2,2,4,4-tetramethylpentane with the active site of the Phenylalanine-tRNA ligase alpha subunit (PheS), in *Bacillus cereus* (UniProt ID: Q633N4) are shown in Fig. 6. 2,2,4,4-Tetramethylpentane interacted with the PheS active site, exhibiting a binding score of ∆G = −4.6 kcal/mol. The 2,2,4,4-tetramethylpentane was tightly tied to the PheS active site through 5 interactions, three of which were Pi–Alkyl bonds, one alkyl, and one Pi–Sigma interaction. 2,2,4,4-Tetramethylpentane binds to protein residues of HIS206, PHE210, and ARG316. Figure 7 illustrate the binding interactions of 2,2,4,4-tetramethylpentane with the active site of the GTPase Der (UniProt ID: P0A6P5) of *Escherichia coli*. 2,2,4,4-Tetramethylpentane showed an interaction with GTPase Der active site binding to protein residues of PRO452, ALA31, and PHE33, achieving a binding score of ∆G = −4.1 kcal/mol. 2,2,4,4-Tetramethylpentane bonded to the GTPase Der active site through 3 interactions: two were alkyl bonds, and one was Pi–Alkyl. 2,2,4,4-Tetramethylpentane binds to protein residues of PRO452, ALA31, and PHE33.

**Fig. 8:**
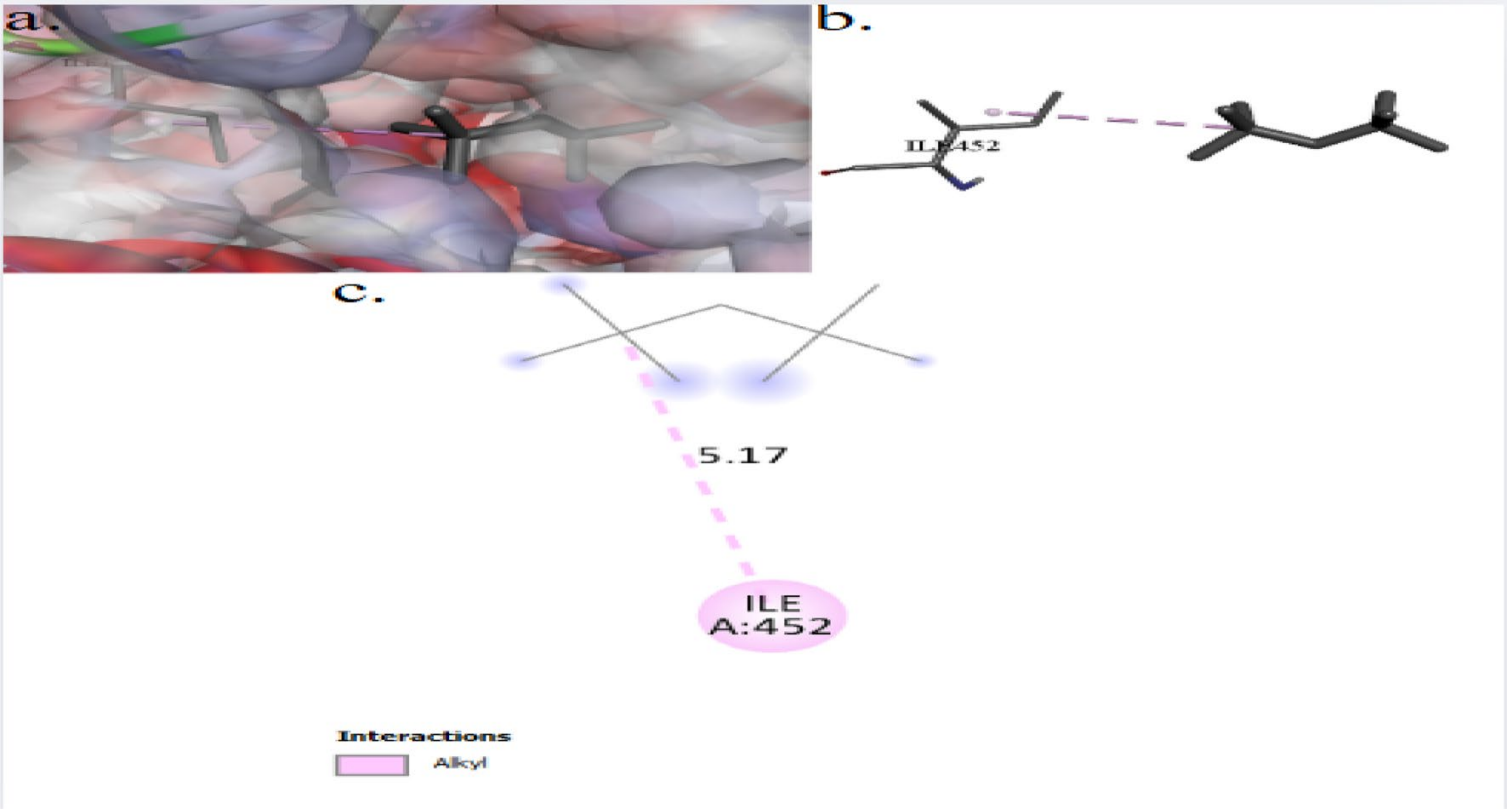
3D (**a**, **b**) and 2D (**c**) molecular docking interaction between the extracted 2,2,4,4-tetramethylpentane and the active site of peptidoglycan-*N*-acetylglucosamine deacetylase (UniProt ID: A0A3Q0NBH7) of *Listeria monocytogenes*

**Fig. 9:**
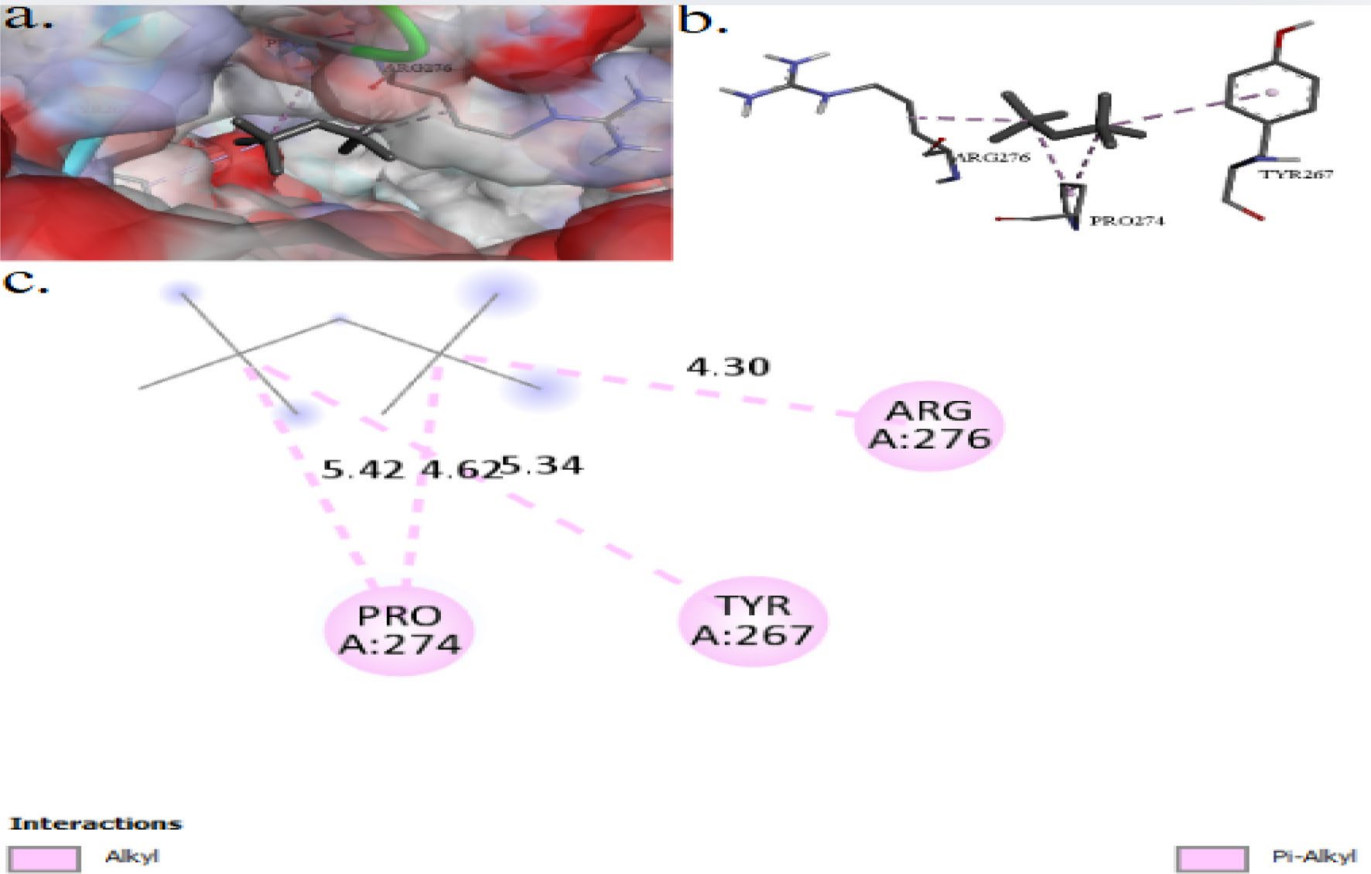
3D (**a**, **b**) and 2D (**c**) molecular docking interaction between the extracted 2,2,4,4-tetramethylpentane and the active site of DNA gyrase subunit B (UniProt ID: P0A2I3) of *Salmonella typhimurium*

The binding interactions of 2,2,4,4-tetramethylpentane with the active site of the peptidoglycan-*N*-acetylglucosamine deacetylase (PgdA) of *Listeria monocytogenes* (UniProt ID: A0A3Q0NBH7) are shown in Fig. 8. 2,2,4,4-Tetramethylpentane showed an interaction with the PgdA active site, achieving a binding score of ∆G = −4.1 kcal/mol. 2,2,4,4-Tetramethylpentane bonded to the protein’s active site through one interaction: alkyl bond with ILE452. In the present study, Fig. 9 illustrates the binding interactions of the tested compound (2,2,4,4-tetramethylpentane) with the active site of the DNA gyrase subunit B of *Salmonella typhimurium* (UniProt ID: P0A2I3). The 2,2,4,4-tetramethylpentane showed an interaction with DNA gyrase subunit B active site, achieving a binding score of ∆G = −4.3 kcal/mol through four interactions: three alkyl bonds and one Pi–Alkyl. 2,2,4,4-Tetramethylpentane binds to protein residues of TYR267, PRO274, and ARG276.

**Fig. 10:**
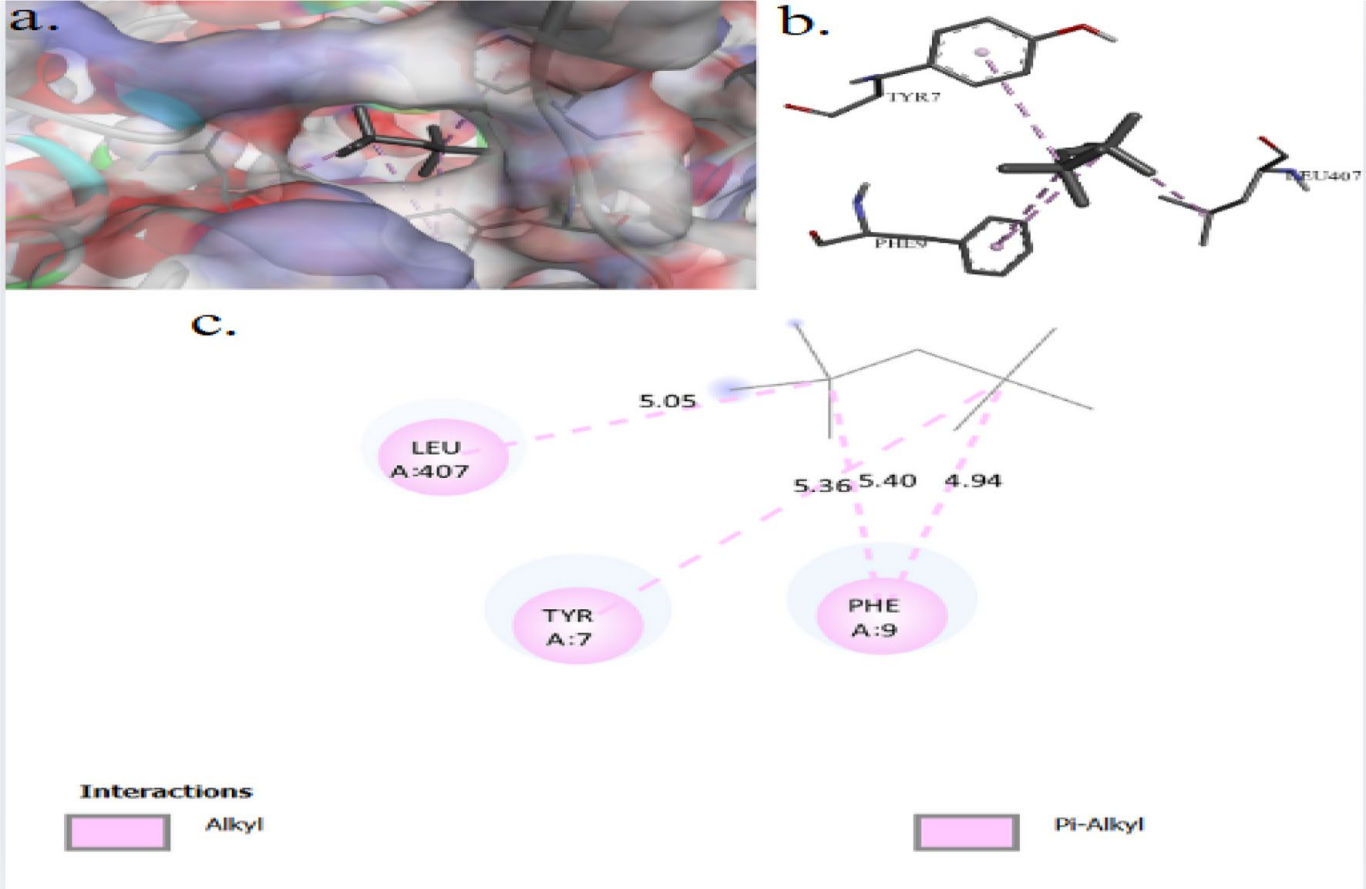
3D (**a**, **b**) and 2D (**c**) molecular docking interaction between the extracted 2,2,4,4-tetramethylpentane and the active site of zinc metalloproteinase aureolysin (UniProt ID: P81177) of *Staphylococcus aureus*

**Fig. 11:**
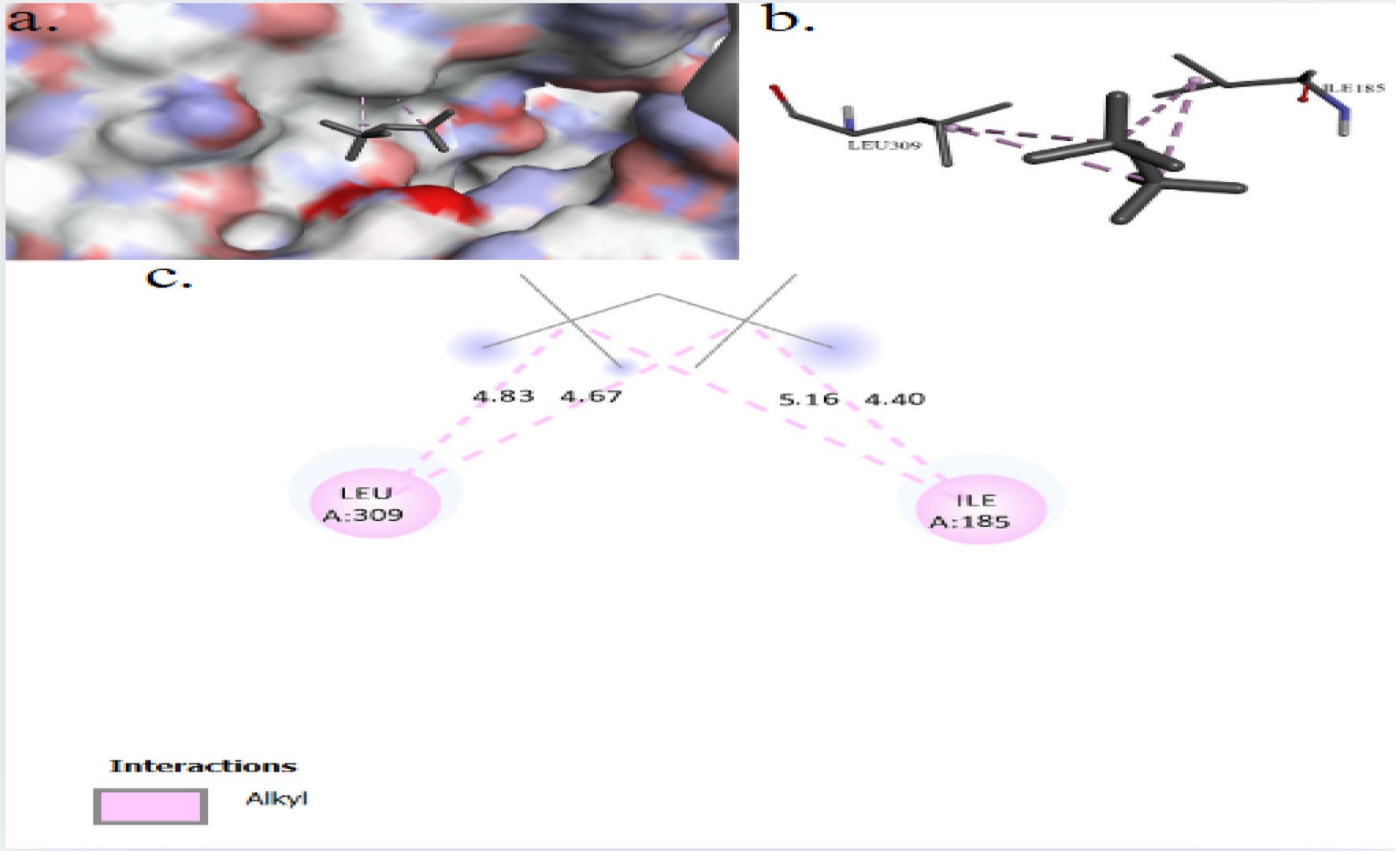
3D (**a**, **b**) and 2D (**c**) molecular docking interaction between the extracted 2,2,4,4-tetramethylpentane and the active site of agglutinin-like protein 2 (UniProt ID: Q9URQ0) of* Candida albicans*

**Fig. 12:**
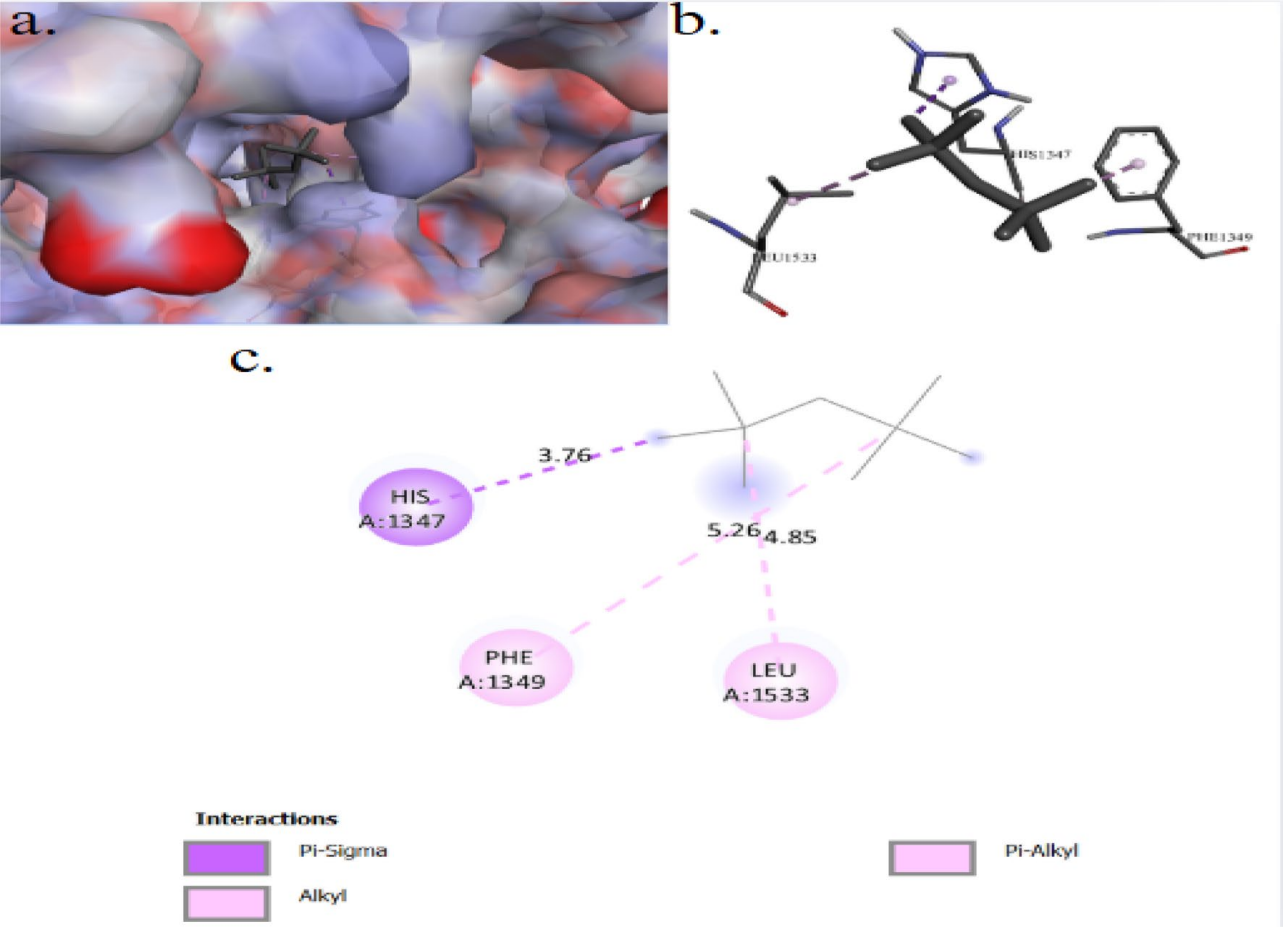
3D (**a**, **b**) and 2D (**c**) molecular docking interaction between the extracted 2,2,4,4-tetramethylpentane and the active site of serine/threonine-protein kinase (UniProt ID: P32600) of *Saccharomyces cerevisiae*

The binding interactions of 2,2,4,4-tetramethylpentane with the active site of the Zinc metalloproteinase aureolysin of *Staphylococcus aureus* (UniProt ID: P81177) are shown in Fig. 10. 2,2,4,4-Tetramethylpentane showed an interaction with the Zinc metalloproteinase aureolysin active site, achieving a binding score of ∆G = −4.7 kcal/mol through four interactions: three Pi–Alkyl bonds and one alkyl bond. 2,2,4,4-Tetramethylpentane binds to protein residues of TYR7, PHE9, and LEU407. Figure 11 illustrate the binding interactions of 2,2,4,4-tetramethylpentane with the active site of the Agglutinin-like protein 2 (ALS2) of *Candida albicans* (UniProt ID: Q9URQ0). 2,2,4,4-Tetramethylpentane showed an interaction with the Agglutinin-like protein 2 active sites, achieving a binding score of ∆G = −3.6 kcal/mol through four interactions: four alkyl bonds with protein residues of ILE185, and ILU309. The binding interactions of 2,2,4,4-tetramethylpentane with the active site of the serine/threonine-protein kinase of *Saccharomyces cerevisiae* (UniProt ID: P32600) are shown in Fig. 12. 2,2,4,4-Tetramethylpentane showed an interaction with the serine/threonine-protein kinase active site, achieving a binding score of ∆G = −3.8 kcal/mol through three interactions: one alkyl bond, one Pi–Alkyl, and one Pi–Sigma. 2,2,4,4-Tetramethylpentane binds to protein residues of LEU1533, PHE1349, HIS1347.

## Discussion

A wide range of endophytic fungi inhabit the internal tissues of plants in a symbiotic relationship and spend their life cycle inside host plants [8]. Endophytic fungi are an important reservoir of secondary metabolites with various biological activities [11]. These metabolites exhibit diverse bioactivities, including antibacterial, anticancer, antidiabetic, antifungal, antiviral, antioxidant, and insecticidal [12]. *C. Procera* is a well-known plant used traditionally to treat Sinus fistulas, skin disease, somatic problems, and diarrhea [20]. In the present study, five fungal species (*Aspergillus flavus*, *A. versicolor*, *A. niger*, *A. versicolor*, and *Rhizopus arrhizus*) accounting for nine colonies were isolated from leaves and stems of *Calotropis procera* from Giza and Cairo in Egypt. *Aspergillus* and *Penicillium* were the most dominant endophytic fungi in medicinal plants inhabiting the Egyptian environment [1]. Mohamed et al. [20] isolated eight fungal species of *Aspergillus niger*, *A. fumigatus*, *A. flavus*, *A. nidulans*, *Fusarium oxysporum*, *Nigrospora oryzae*, *Penicillium chrysogenum* and *Trichoderma harzianum* from *C. procera*. Four endophytic fungi (*Aspergillus* sp., *Alternaria* sp., *Cladosporium* sp., and *Mycelia sterilia* sp.) from *C. procera* were isolated by Khiralla et al. [36] who indicated that the low diversity of the endophytes of the medicinal plants might be due to the significant variation in climatic conditions such as rainfall and atmospheric humidity.

The tested endophytic fungal crude extract from *C. procera* exhibited antimicrobial potential against seven pathogenic microorganisms, three gram-positive bacteria, including *Bacillus cereus* (EMCC 1080), *Staphylococcus aureus* (ATCC 25923), and *Listeria monocytogenes* (ATCC 7644), two gram-negative bacteria include *Escherichia coli* (wild type strain 93111), and *Salmonella typhimurium* (ATCC 14028), and two fungal species include *Saccharomyces cerevisiae* (ATCC 9763) and *Candida albicans* (EMCC 105). Ethyl acetate and chloroform extract of isolated endophytic fungi showed prominent antimicrobial potential in the inhibition zone against tested bacterial and fungal species. Farouk et al. [1] indicated that the endophytes of medicinal and edible plants might be an antibiotic source to control pathogens. Endophytic fungi can produce a wide range of bioactive antimicrobial compounds that inhibit the growth of phytopathogens [37]. Endophytes are a prospective source of unique bioactive pharmacological compounds, as these strains can synthesize secondary metabolites that safeguard plants from pathogens and insects [38]. Ethyl acetate extract of isolated endophytic fungi showed the highest antimicrobial potential against tested species. Our finding agrees with Garcia et al. [39], who revealed that the ethyl acetate solvent system was an extremely efficient strategy to extract principal endophytic fungal compounds with excellent antibacterial activity compared to other solvents.

Ethyl acetate extract of endophytic *Aspergillus flavus* showed the highest clear inhibition zone (31 mm) against *S. typhimurium*, followed by *B. cereus* (20 mm). In comparison, the chloroform extract of *A. flavus* was effective against *S. typhimurium* showing an inhibition zone of 30 mm. The endophytic fungus *Aspergillus nomius* extract obtained from *C. procera* presented antibacterial activity against *Salmonella typhi* [23]. The endophytic fungal extract causes loss of structural integrities in bacteria, causing rupture, degeneration, and deformation of cell structures [29]. Extensive degradation and cellular shrinkages revealed rupture of the cell membrane and loss of intracellular contents after treatment [40]. Ethyl acetate extract may directly or indirectly interfere with the metabolic energy of examined bacterial cells via core carbohydrate metabolic pathways [41]. The potent inhibitory activities of antifungal metabolites produced by the endophytic isolate resulted in the diminishing of the conidial development and germ tube elongation of *P. digitatum* in the presence of lower doses of ethyl acetate extract [42].

Ethyl acetate extract of endophytic *Aspergillus niger* exhibited the highest antibacterial activity against *L. monocytogenes* with an inhibition zone of 24 mm. Furthermore, the chloroform extract of *A. niger* showed the highest anticandidal and antibacterial activity against *C. albicans* (26 mm) and *S. aureus* (24 mm). The antibacterial compounds extracted from endophytic fungus derived from medicinal exhibited deformation of *Klebsiella pneumonia* and *E. coli* with several pore formations [43]. In addition, ethyl acetate extract of endophytic fungal species displayed anticandidal activity against *C. albicans*, causing its destruction observed by scanning electron microscopy. At the same time, fluconazole, the widely employed antifungal drug, exhibited a static mode of action against *C. albicans* [44].

Chloroform extract of endophytic *Aspergillus fumigatus* showed the highest antibacterial activity against *L. monocytogenes* (25.33 mm). In comparison, the highest inhibition zone (26 mm) of *S. cerevisiae* was exhibited by chloroform extract of endophytic *Aspergillus versicolor*. The activity of endophytic fungal extract may be due to total phenolic content as recorded by Khiralla et al. [36], who found that ethyl extract of endophytic *Aspergillus* sp. from *C. procera* seed contains a high content of low and high molecular weight polyphenols, which have antimicrobial and antioxidant activities. The ethyl acetate extract of *Aspergillus clavatonanicus* isolated from *Mirabilis jalapa* provided considerable suppression of the radial growth of *Fusarium graminearum*, *F. culmorum*, and *F. oxysporum* [45]. In the present investigation, *S. typhimurium* was more resistant to ethyl acetate and chloroform extract of *Rhizopus arrhizus*, potentially attributable to their fundamental variation in the cell wall properties. In gram-negative bacteria, the outer membrane serves as a cell’s permeability barrier, preventing the entry of lipophilic solutes [46].

The ethyl acetate extracts of the five endophytic fungal species exhibited in vitro cytotoxicity activity against the two cancer cell lines: MCF-7 (Breast cancer) and HCT-16 (Colon Cancer). At the same time, BHK was used as a standard cell line. Fungal endophytes produce a wide range of natural compounds, including secondary metabolites with anticancer properties, making them a valuable resource for drug discovery [47]. The crude extract of the endophytic fungus *Aspergillus niger* exhibited the highest anticancer activity against MCF-7 and HCT-16 with an IC50 value of 8.5 and 17.8 µg/mL, respectively. The ethyl acetate extract of the endophytic *Aspergillus fumigatus* displayed strong cytotoxic activity against HCT-116, exhibiting an IC50 value of 7.8 µg/mL. On the other hand, crude extracts of *Aspergillus flavus* and *Rhizopus arrhizus* exhibited cytotoxic effects against cancer cells, but only at concentrations toxic to normal line cells. Secondary metabolites from endophytes associated with medicinal plants displayed remarkable anticancer activity against human cancer cell lines [48]. The crude extract of the endophytic *Cladosporium cladosporioides* demonstrated anticancer activity against cell lines (HL-60, A375, PC-3, and MCF-7), with particularly potent efficacy against PC-3, revealed an IC50 value of 0.74 µg/mL. The extract of *Lasiodiplodia theobromae* displayed anticancer activity against three cell lines: IC50 value = 1.19 µg/mL against MCF-7, IC50 value = 1.045 µg/mL against HCT-116, and IC50 value = 39.73 µg/mL against PC-3. The crude extract of endophytic *Talaromyces purpureogenus* exhibited potent cytotoxic activity against HCT-116 with an IC50 value of 1.51 µg/mL [11]. In our study, *Aspergillus niger* was chosen for further investigation due to its superior antimicrobial and anticancer activities. Alignment of sequences of the tested isolated *Aspergillus niger* based on their ribosomal internal transcribed spacer (ITS) sequences showed 100% sequence homology with *A. niger* isolate cu-17 (MT536778.1) from the gene bank based on the phylogenetic analysis, and it has accession number PQ568010.

The ethyl acetate extract of the tested isolated *Aspergillus niger* was characterized and identified by GC/MS analysis. GC/MS analysis revealed that the fungal endophyte *Aspergillus niger* can synthesize a variety of bioactive compounds. Various types and numbers of compounds were detected by GC–MS study. *A. niger* was the most effective endophytic fungal species, which synthesizes secondary metabolites, including polyethylene polymer, 2,2,4,4-tetramethyl pentane (Nonane), propyl hexadecanoate (propyl palmitate), and stearamide. Tetramethyl pentane and propyl hexadecanoate have antimicrobial activities as recorded by Kumari et al. [49] who showed that secondary metabolites (3-methylpentane, cyclohexadecane, linoleic acid, Hexadecane, Heptadecane, Nonadecane, and tricontane) from rhizospheric actinomycetes have been recognized to obtain antimicrobial, antitumor, antidiabetic, anti-inflammatory activities. GC/MS analysis revealed that fungal endophyte *Penicillium Singorense* can produce a variety of bioactive compounds, including 1-docosene, 1-heneicosanol 1-nonadecene, 1-octadecene, 3-eicosene, *E*-15-heptadecenal, cyclohexadecane, cyclopentasiloxane, and eicosyl methyl ether. Eicosene and nonadecene have antimicrobial activity [50]. GC–MS analysis of rhizospheric actinomycetes isolates identified dibutyl phthalate, *N*-hexadecanoic acid, 1-nonadecene, 3,7,11,15-tetramethyl-2-hexadecene, Hexadecanoic acid, and 2,3-dihydroxypropyl ester as major compounds with antimicrobial potential. These compounds exhibited activity against *Staphylococcus aureus* MTCC-3160 (IZ = 18–25 mm), *Pseudomonas aeruginosa* MTCC 1688 (IZ = 11–24 mm), *Klebsiella pneumonia* MTCC-432 (IZ = 19–34 mm), *Proteus vulgaris* MTCC-7306 (IZ = 10–30 mm), *Bacillus subtilis* MTCC-441 [51]. Stearamide extracted from *Solanum schimperianum* showed antimicrobial activity against *E. coli* and *Candida albicans* [52].

The antimicrobial metabolites from the ethyl acetate extract of *A. niger* were separated and partially purified using the TLC method. The ethyl acetate extract of *A. niger* showed two bands with different Rf values, including band 1 (R_f_ = 2 cm) and band 2 (R_f_ = 3 cm) under UV light. Band 2 (R_f_ = 3 cm) showed inhibitory activity against gram-positive and gram-negative bacteria. The extract of *Alternaria tenuissima*, obtained from the root of *Salvia przewalskii*, a traditional Chinese medicinal plant, produces three novel pyrones [53]. Qader et al. [54] investigated the antimicrobial efficacy of the pure compounds derived from isolated endophytic fungi against pathogenic gram-positive bacteria (*Staphylococcus aureus* and *Bacillus subtilis*), gram-negative bacteria (*Escherichia coli*, *Klebsiella pneumoniae*, *Proteus vulgaris*, and *Pseudomonas aeruginosa*) and yeast (*Candida albicans*). The antimicrobial activity of these compounds varied against different bacteria and *C. albicans*.

^1^H NMR analyses of the cell-free extract of *A. niger* from band 2 of TLC produce one antimicrobial compound with the molecular formula is C₉H₂₀. A potential candidate is 2,2,4,4-tetramethylpentane (a highly branched alkane) (Fig. 6). Elgorban et al. [55] identified 62 biologically active compounds in the ethyl acetate extracts of an endophytic fungus *Alternaria* sp. isolated from *Salvadora persica* in Saudi Arabia. The compound ethyl 4-{[*N*-(2,2,4,4-Tetramethylchroman-6-yl)thiocarbamoyl]amino}benzoate exhibited promising antibacterial activity, with MIC values of 5.0–10.0 µg/mL. Its mechanism of action is speculated to involve the inhibition of mycolic acid synthesis and/or dihydrofolate reductase, an essential enzyme for folate metabolism in both eukaryotic and prokaryotic cells [56].

Modeling studies were conducted to elucidate the binding model and interaction of the bioactive compounds from the potent endophytic fungal extract (2,2,4,4-tetramethylpentane) within the target protein’s active site. Accordingly, molecular docking studies were carried out against five bacterial proteins and two fungal proteins, including Phenylalanine-tRNA ligase alpha subunit (UniProt ID: Q633N4), GTPase Der (UniProt ID: P0A6P5), peptidoglycan-*N*-acetylglucosamine deacetylase (UniProt ID: A0A3Q0NBH7), DNA gyrase subunit B (UniProt ID: P0A2I3) and Zinc metalloproteinase aureolysin (UniProt ID: P81177), which are expressed for *Bacillus cereus*, *Escherichia coli*, *Listeria monocytogenes*, *Salmonella typhimurium* and *Staphylococcus aureus*, respectively. However, Agglutinin-like protein 2 (UniProt ID: Q9URQ0) and serine/threonine-protein kinase (UniProt ID: P32600) are expressed for *Candida albicans* and *Saccharomyces cerevisiae*, respectively. Proteins were optimized by eliminating water molecules, adding polar hydrogens, and assigning Gasteiger charges. The bond order of the ligand and protein was adjusted. The bioactive compound from the potent endophytic fungal extract was obtained from the PubChem database in SDF format and then subjected to energy minimization by Avogadro software.

2,2,4,4-Tetramethylpentane interacted with the Phenylalanine-tRNA ligase alpha subunit (PheS) active site in *Bacillus cereus*, exhibiting a binding score of ∆G = −4.6 kcal/mol. The 2,2,4,4-tetramethylpentane was tightly tied to the PheS active site through 5 interactions, three of which were Pi–Alkyl bonds, one alkyl, and one Pi–Sigma interaction. 2,2,4,4-Tetramethylpentane binds to protein residues of HIS206, PHE210, and ARG316. The 2,2,4,4-tetramethylpentane can potentially disrupt the binds to PheS’s function and prevent the enzyme from esterifying phenylalanine to tRNA^*Phe*^, leading to significant effects on bacterial protein synthesis as recorded by Garcia-Rubio et al. [57] who stated that the PheS facilitates the esterification of phenylalanine to its corresponding tRNA (tRNA^*Phe*^), thereby ensuring accurate translation of the genetic code in *B. cereus*. This process is vital for protein synthesis, providing phenylalanyl-tRNA for ribosomal incorporation into elongating polypeptides. Also, aminoacylated tRNAs are crucial for accurate translation of the genetic code, as any errors in matching tRNAs with their corresponding amino acids may result in mistakes in protein synthesis [58].

The binding interactions of 2,2,4,4-tetramethylpentane with the active site of the GTPase Der of *Escherichia coli*. 2,2,4,4-Tetramethylpentane showed an interaction with GTPase Der active site binding to protein residues of PRO452, ALA31, and PHE33, achieving a binding score of ∆G = −4.1 kcal/mol. 2,2,4,4-Tetramethylpentane bonded to the GTPase Der active site through 3 interactions: two were alkyl bonds, and one was Pi–Alkyl. 2,2,4,4-Tetramethylpentane binds to protein residues of PRO452, ALA31, and PHE33. The GTPase Der plays a crucial role in the biogenesis and stability of 50S ribosomal subunits, ensuring proper ribosome assembly and function. It interacts with 50S subunits in a GTP-dependent manner, influencing rRNA processing and ribosomal protein incorporation [59]. The involvement of The GTPase Der is crucial for the synthesis of the 50S ribosome, which is vital for cellular survival and proliferation, highlighting its essential role in maintaining cellular functions [60].

2,2,4,4-Tetramethylpentane showed an interaction with the peptidoglycan-*N*-acetylglucosamine deacetylase (PgdA) active site of *Listeria monocytogenes*, achieving a binding score of ∆G = −4.1 kcal/mol. 2,2,4,4-Tetramethylpentane bonded to the protein’s active site through one interaction: alkyl bond with ILE452. The PgdA in *L. monocytogenes* is essential for bacterial proliferation and division, providing resistance to cell wall hydrolases, mutanolysin, and peptide antibiotics (colistin) [61]. It also safeguards cells from autolysis induced by lysozyme or other autolysis-inducing agents [62].

The 2,2,4,4-tetramethylpentane showed an interaction with DNA gyrase subunit B active site of *Salmonella typhimurium*, achieving a binding score of ∆G = −4.3 kcal/mol through four interactions: three alkyl bonds and one Pi–Alkyl. 2,2,4,4-Tetramethylpentane binds to protein residues of TYR267, PRO274, and ARG276. DNA gyrase is a vital topoisomerase in bacteria that is crucial for managing nucleoid structure, DNA replication, transcription, and recombination. It is also a significant drug target [63]. DNA gyrases and topoisomerase IV are essential for maintaining chromosomal metabolism in bacteria [64].

2,2,4,4-Tetramethylpentane showed an interaction with the Zinc metalloproteinase aureolysin active site of *Staphylococcus aureus*, achieving a binding score of ∆G = −4.7 kcal/mol through four interactions: three Pi–Alkyl bonds and one alkyl bond. 2,2,4,4-Tetramethylpentane binds to protein residues of TYR7, PHE9, and LEU407. The Zinc metalloproteinase aureolysin contributes to the survival and virulence of *Listeria monocytogenes* by inhibiting the deposition of host C3b on bacterial surfaces and reducing immune cell recruitment and pathogen recognition [65]. Aureolysin may further modulate immunological responses by affecting the activation of T and B lymphocytes and suppressing of lymphocyte immunoglobulin production [66]. The binding interactions of 2,2,4,4-tetramethylpentane with the active site of the Agglutinin-like protein 2 (ALS2) of *Candida albicans*, achieving a binding score of ∆G = −3.6 kcal/mol through four interactions: four alkyl bonds with protein residues of ILE185, and ILU309. ALS2 facilitates the attachment of *Candida albicans* to host tissues and adhering to abiotic surfaces, establishing infections and initiating host–pathogen interactions [67].

The binding interactions of 2,2,4,4-tetramethylpentane with the active site of the serine/threonine-protein kinase of *Saccharomyces cerevisiae*, achieving a binding score of ∆G = −3.8 kcal/mol through three interactions: one alkyl bond, one Pi–Alkyl, and one Pi–Sigma. 2,2,4,4-Tetramethylpentane binds to protein residues of LEU1533, PHE1349, HIS1347. The Target of Rapamycin Complex 2 (TORC2) is a serine/threonine protein kinase complex highly conserved across eukaryotes. In *Saccharomyces cerevisiae*, TORC2 is an essential regulator of cellular processes linked to plasma membrane homeostasis, ensuring the cell membrane’s proper structure and function. In this role, TORC2 regulates a variety of cellular processes, including sphingolipid synthesis, glycerol production and efflux, the actin cytoskeleton polarization, and endocytosis (68).

## Conclusion

There is an urgent need for safer, sustainable, and environmentally friendly antimicrobial agents derived from natural sources. The present study evaluated the capability of endophytic fungi as a source of novel bioactive compounds with antimicrobial and anticancer properties. Characterization of active metabolites suggested the isolate’s production of several bioactive compounds. The most potent endophytic fungal species was *A. niger*, which synthesizes secondary metabolites, including polyethylene polymer, 2,2,4,4-tetramethyl pentane (Nonane), propyl hexadecanoate (propyl palmitate), and stearamide. Although one compound has been identified from the active fractions, further purification steps are necessary to get the purified active compounds, which will aid in developing targeted drugs. Overall finding strongly suggested that the strain *Aspergillus niger* is a valuable source of bioactive metabolites, holding potential for the development of effective drugs to benefit human health.

## Data Availability

Data is provided within the manuscript.

## Materials availability

The materials used in this study are available from the corresponding author upon reasonable request.

## Code availability

No code was generated or used during the current study.
